# Krüppel-like Factor 11 Regulates the Expression of Metabolic Genes via an Evolutionarily Conserved Protein Interaction Domain Functionally Disrupted in Maturity Onset Diabetes of the Young*[Fn FN2]

**DOI:** 10.1074/jbc.M112.434670

**Published:** 2012-04-15

**Authors:** Gwen Lomberk, Adrienne Grzenda, Angela Mathison, Carlos Escande, Jin-San Zhang, Ezequiel Calvo, Laurence J. Miller, Juan Iovanna, Eduardo N. Chini, Martin E. Fernandez-Zapico, Raul Urrutia

**Affiliations:** From the ‡ Laboratory of Epigenetics and Chromatin Dynamics, Epigenomics Translational Program, Mayo Clinic Center for Individualized Medicine, Division of Gastroenterology and Hepatology, and Department of Biochemistry and Molecular Biology,; § Department of Anesthesiology and Kogod Aging Center, and; ¶ Schulze Center for Novel Therapeutics, Division of Oncology Research, Mayo Clinic, Rochester, Minnesota 55905,; ‖ Molecular Endocrinology and Oncology Research Center, CHUL Research Center, Quebec 61V 462, Canada,; ** Department of Molecular Pharmacology and Experimental Therapeutics, Mayo Clinic, Scottsdale, Arizona 85259, and; ‡‡ Centre de Recherche en Carcérologie de Marseille, INSERM UMR 1068, CNRS UMR 7258, Aix-Marseille University and Institut Paoli-Calmettes, Parc Scientifique et Technologique de Luminy, Marseille F-13288, France

**Keywords:** G Proteins, Gene Regulation, Krüppel-like Factor (KLF), Protein-Protein Interactions, Transcription, Gβ_2_, KLF11, WD40, Metabolic Gene Networks

## Abstract

The function of Krüppel-like factor 11 (KLF11) in the regulation of metabolic pathways is conserved from flies to human. Alterations in KLF11 function result in maturity onset diabetes of the young 7 (MODY7) and neonatal diabetes; however, the mechanisms underlying the role of this protein in metabolic disorders remain unclear. Here, we investigated how the A347S genetic variant, present in MODY7 patients, modulates KLF11 transcriptional activity. A347S affects a previously identified transcriptional regulatory domain 3 (TRD3) for which co-regulators remain unknown. Structure-oriented sequence analyses described here predicted that the KLF11 TRD3 represents an evolutionarily conserved protein domain. Combined yeast two-hybrid and protein array experiments demonstrated that the TRD3 binds WD40, WWI, WWII, and SH3 domain-containing proteins. Using one of these proteins as a model, guanine nucleotide-binding protein β2 (Gβ_2_), we investigated the functional consequences of KLF11 coupling to a TRD3 binding partner. Combined immunoprecipitation and biomolecular fluorescence complementation assays confirmed that activation of three different metabolic G protein-coupled receptors (β-adrenergic, secretin, and cholecystokinin) induces translocation of Gβ_2_ to the nucleus where it directly binds KLF11 in a manner that is disrupted by the MODY7 A347S variant. Using genome-wide expression profiles, we identified metabolic gene networks impacted upon TRD3 disruption. Furthermore, A347S disrupted KLF11-mediated increases in basal insulin levels and promoter activity and blunted glucose-stimulated insulin secretion. Thus, this study characterizes a novel protein/protein interaction domain disrupted in a KLF gene variant that associates to MODY7, contributing to our understanding of gene regulation events in complex metabolic diseases.

## Teaser

**Background:** Most genetic alterations in MODY affect transcription factors.

**Results:** We functionally characterize a conserved domain in KLF11, which is disrupted in MODY7 patients.

**Conclusion:** Defects in KLF11 not only affect insulin biosynthesis but also diabetes-associated metabolic gene networks.

**Significance:** Disruption of this KLF11 regulatory domain as in MODY7 has wider effects on β cell gene expression than anticipated, helping to clarify disease mechanisms.

## Introduction

Krüppel-like factor 11 (KLF11),^3^ a human ortholog of the *Drosophila* gene *cabut*, belongs to the KLF family of transcription factors. Members of this family regulate GC promoters in organisms ranging from flies to humans (1). Rapidly emerging evidence demonstrates that these *cabut*/KLF pathways also regulate important metabolic processes conserved in organisms ranging from flies to humans (2). For instance, *cabut* is a transcriptional regulator of metabolic gene pathways in *Drosophila* (3, 4). Disruption of KLF pathways leads to biochemical alterations and metabolic impairment, often resulting in lethality. In fact, human variants in the KLF11 protein and its DNA binding site within the insulin promoter cause MODY7 and neonatal diabetes, respectively (5, 6). Moreover, extensive studies have demonstrated that KLF11 binds and regulates many promoters of genes involved in cholesterol, prostaglandin, neurotransmitter, fat, and sugar metabolism (5–11). Thus, the medical significance of this knowledge led us to study how alterations in KLF11 proteins impact the regulation of metabolic gene networks of relevance to gain a better understanding of complex human diseases.

KLF11 is a well characterized protein in terms of its ability to couple to several chromatin pathways and epigenetic regulators, including Sin3a, histone acetyltransferase, and HP1 (12–14). The following study was designed to further our mechanistic understanding of how disease-associated alterations affect the membrane-to-nucleus coupling of KLF11 to the regulation of metabolic genes. Contrary to the variants in KLF11 DNA binding sites, which have the power to uncouple a single gene promoter (*e.g.* c.-331 *INS* variants) (5), alterations in chromatin coupling can disrupt complex functions, such as metabolism, by altering the expression of entire gene networks. Interestingly, variants that fall near the Sin3a domain alter the ability of KLF11 to regulate metabolic target genes and associate with MODY7 (6). The KLF11-mediated histone acetyltransferase pathways appear to impact the regulation of insulin in neonatal diabetes (5). However, the mechanisms and function of other KLF11 mutations that associate with diseases remain to be characterized. Thus, studying these types of alterations can aid in better defining the identities of KLF11-mediated metabolic gene networks, the mechanisms of their regulation, and the mechanisms that inactivate these pathways in humans.

The KLF11 A347S variant found in MODY7 diabetic patients (6) maps to the previously characterized transcriptional regulatory domain 3 (TRD3) for which functional cofactors have remained unknown (15). In the current study, we demonstrate that the KLF11 TRD3 functions as a novel protein/protein interaction domain and that its function is altered in the A347S diabetes variant. We further show that activation of cell surface receptors involved in metabolism (β-adrenergic, secretin, and cholecystokinin (CCK) receptors) can induce the translocation of a TRD3-binding protein (Gβ_2_) to the nucleus where it binds KLF11 to regulate metabolic gene targets. Combined, these studies reveal the existence of a novel KLF-mediated pathway for coupling extracellular signals to the regulation of gene expression related to metabolism and diabetes. These findings extend our understanding of transcriptional regulatory events that are disrupted in human metabolic diseases.

## EXPERIMENTAL PROCEDURES

### 

#### 

##### Tissue Culture and Reagents

Cell lines were obtained from the American Type Culture Collection (ATCC, Manassas, VA). Cells were cultured as described previously (16, 17). CHO cells stably transfected with CCK receptor A (CHO-CCKAR) were grown as described previously (18). Isoproterenol, secretin, and CCK were purchased from Sigma-Aldrich.

##### Plasmids and Recombinant Adenovirus

Standard molecular biology techniques were used to clone full-length KLF11, KLF11 A347S, and Gβ_2_ as well as specified deletions into pcDNA3.1/His (Invitrogen), pCMV-Tag 2B (Stratagene, La Jolla, CA), pGEX (GE Healthcare), and EYFP vectors as described previously (14, 19). QuikChange® site-directed mutagenesis was performed as suggested by the manufacturer (Agilent Technologies, Inc., Santa Clara, CA). All constructs were verified by sequencing at the Mayo Clinic Molecular Biology Core Facility. Epitope-tagged (His_6_ Xpress^TM^) KLF11 and KLF11 A347S variant as well as empty vector (Ad5CMV) were generated as recombinant adenovirus in collaboration with the Gene Transfer Vector Core at the University of Iowa.

##### GST Pulldown Assays, Immunoprecipitation, and Western Blot

GST and GST fusion protein purification, *in vitro* translation, GST pulldown assays, immunoprecipitation, and Western blot were all done as described previously (19). Antibodies against the FLAG (Sigma) or His tag (OMNI D8, Santa Cruz Biotechnology, Santa Cruz, CA) were used to detect recombinant expression of KLF11, KLF11 A347S variant, and Gβ_2_; endogenous Gβ_1/2_ (Santa Cruz Biotechnology); HP1α (Millipore, Billerica, MA); Sin3a (Santa Cruz Biotechnology); or CBP (Abcam, Cambridge, MA).

##### Algorithm for Identifying TRD3

To identify putative evolutionarily conserved domains, the N termini prior to the conserved zinc finger regions of cabut (*Drosophila*) and KLF1–17 (*Homo sapiens*) were analyzed using a sliding window of 15 amino acids. Windows were scored for overall hydrophobicity using the Kyte-Doolittle scale (20); polyproline helix conformations using experimentally derived values (21); and overall proline, glycine, and glutamine content (PGQ index). Regions were screened via pairwise alignment with KLF10 and KLF11 for R1 and R2 domains to determine the N-terminal boundary. Pairwise alignments were performed using the Multiple Sequence Comparison by Log-Expectation Program (MUSCLE) (22). A phylogenetic tree was constructed using Geneious Tree Maker (Biomatters Ltd., Auckland, New Zealand) with the following settings: BLOSUM62 matrix, global alignment with free gap ends, and outgroup set to cabut.

##### Identification of TRD3-binding Proteins Using Yeast Two-hybrid System

As bait, the coding region of the KLF11 TRD3 (amino acids 273–351) was cloned into the pGBKT7 plasmid of the Matchmaker system (Clontech). The integrity and expression of the fusion construct were confirmed by sequencing. No autoactivation of the reporters was associated with this bait construct as determined by cotransformation of the bait with prey library vector in host AH109 cells. Sequential library scale transformations were performed from a normal bone marrow cDNA library. A total of 2.5 million clones were screened and selected on high stringency plates (Synthetic Defined medium/−Ade/−His/−Leu/−Trp) coated with 5-bromo-4-chloro-3-indolyl-β-d-galactopyranoside. After incubation for a period of 72–96 h at 30 °C, colonies were recovered, and DNA from each colony was extracted and sequenced. The cDNA inserts from yeast clones were amplified by PCR using primers 5′-CTATTCGATGATGAAGATACCCCACCA-3′ (forward) and 5′-GTGAACTTGCGGGGTTTTTCAGTATCTACGA-3′ (reverse) and sequenced at the Mayo Molecular Biology Core Facility. To eliminate false positives, isolated library prey plasmids were transformed into Y187 yeast and crossed with AH109 yeast carrying either the empty plasmid or the bait plasmid; activation of the reporter gene was assessed by growth in Synthetic Defined medium/−Trp/−Leu/−His plus 3-amino-1,2,4-triazole.

##### Identification of TRD3-binding Proteins Using Solid Phase Binding Assays

To screen for binding between the KLF11 TRD3 and SH3 or WW domain-containing proteins, solid phase SH3 and WW domain arrays were obtained from Panomics (Fremont, CA) and processed according to the manufacturer's instructions utilizing 10–20 μg/ml purified His-KLF11 TRD3 (amino acids 273–351) for hybridization.

##### Immunofluorescence and Bimolecular Fluorescence Complementation (BiFC)

CHO-CCKAR cells were transfected with FLAG-tagged or His-tagged Gβ_2_ and KLF11 expression constructs. The fluorescence was imaged using an LSM510 microscope (Zeiss, Heidelberg, Germany). For the BiFC analysis of individual cells by confocal microscopy, HeLa and Capan2 cells were transfected with KLF11-EYFP1 and Gβ_2_-EYP2 expression vectors as described previously (13, 19).

##### Genome-wide Expression Profiles

Pancreatic cells (rat INS-1 β cells (multiplicity of infection, 10:1) and human Panc1 pancreatic adenocarcinoma cells (multiplicity of infection, 150:1)) were plated at a density of 10^6^ cells and transduced with empty vector, KLF11, or KLF11 A347S (Ad5CMV). RNA was prepared as described previously (23). Global gene expression profiling was carried out at the Microarrays Facility of the Research Center of Laval University CRCHUL utilizing the Affymetrix Human Gene 1.0 ST arrays (28,869 well annotated genes and 764,885 distinct probes). Intensity files were generated by Affymetrix GCS 3000 7G and the Gene-Chip Operating Software (Affymetrix, Santa Clara, CA). Data analysis, background subtraction, and intensity normalization were performed using robust multiarray analysis (24). Genes that were differentially expressed and the false discovery rate were estimated from a *t* test (<0.005) and corrected using the Bayes approach (25, 26). Data analysis, hierarchical clustering, and ontology were performed with the OneChanelGUI to extend affylmGUI graphical interface capabilities (27) and Partek Genomics Suite, version 6.5 (Partek Inc., St. Louis, MO) with analysis of variance analysis. The criteria of log_2_ -fold change ±1.5 and a *p* value of <0.05 compared with empty vector control or wild type KLF11 levels were used to determine significant gene targets. A subset of genes was validated by quantitative PCR as described previously (23, 28). The criteria of log_2_ -fold change ±1.5 and a *p* value of <0.05 compared with empty vector control were used to determine significant gene targets. HeLa cells were plated at a density of 10^6^ cells and transfected with empty vector, Gβ, or Gβ fused in-frame to three copies of the SV40 nuclear localization signal (29). RNA was prepared as described above. Global gene expression profiling was carried out by the Microarray Core of Mayo Clinic utilizing the Affymetrix Human Genome U133 Plus 2.0 Array. The criteria of log_2_ -fold change ±1.5 and a *p* value of <0.05 compared with empty vector control were used to determine significant gene targets.

##### Chromatin Immunoprecipitation (ChIP)

ChIP was performed as described previously (13, 16, 19, 28) using antibodies against His tag (OMNI D8, Santa Cruz Biotechnology) to detect recombinant expression of KLF11 or endogenous Gβ_1/2_ (Santa Cruz Biotechnology). Binding activity was derived using the NimbleGen human promoter hybridization system (Madison, WI). Peaks were detected by searching for >4 probes where signals were above the specified cutoff values (90–15%) using a 500-bp sliding window along 5 kb upstream of the transcriptional start site in human promoters. Each peak was assigned a score that is the log_2_ ratio of the fourth highest probe in each peak. If multiple peaks are present, the peak nearest the transcription start site is reported. Ratio data were then randomized 20 times to evaluate the false discovery rate. Only peaks with false discovery rate scores <0.2 were deemed high confidence binding sites and reported.

##### Glucose-stimulated Insulin Secretion and Insulin Promoter-Luciferase Assays

INS-1 cells were transduced with virus (empty vector, KLF11, or KLF11 V347; multiplicity of infection, 10:1) for 48 h prior to insulin measurements. Cells were incubated in standard growth medium prior to initiation of the time course experiment. Medium was replaced with Krebs buffer (no glucose) with samples obtained at 0, 15, 30, 45, and 60 min. After 60 min without glucose, cells were stimulated by the addition of glucose (10 mm), and samples were collected at 0 and 120 min. Measurement of secreted insulin was completed with the ultrasensitive Rat Insulin ELISA kit (Crystal Chem, Downers Grove, IL). The *INS* wild type promoter-reporter construct and luciferase assays were as described previously (5). Each experiment was performed at least three different times in triplicate, data were expressed as mean ± S.E., and statistical analyses were performed using Student's *t* test.

## RESULTS

### 

#### 

##### The KLF11 A347S Variant Maps to a Protein Interaction Domain Conserved in the KLF Family of Metabolic Transcription Factors

**FIGURE 1.**
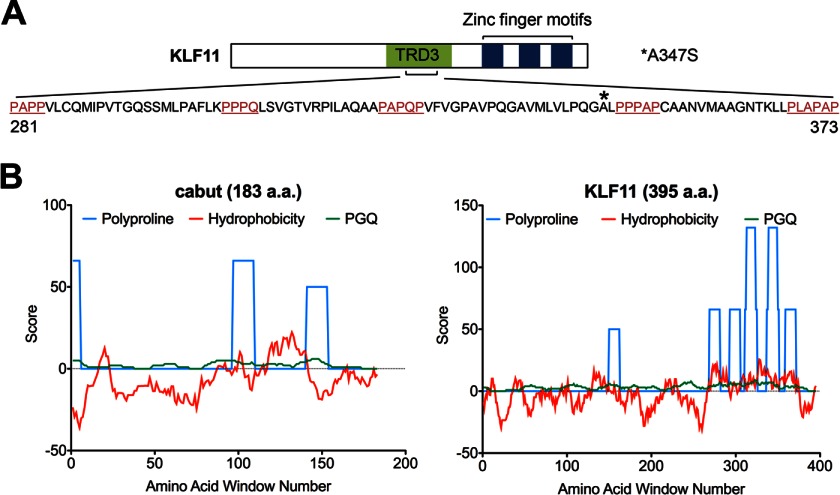
**KLF11 A347S variant maps to a novel hydrophobic glycine-glutamine-proline-rich domain that is observed in the corresponding region of its fly ortholog *cabut*.** *A*, diagrammatic representation of the location and composition of a conserved protein-to-protein interaction domain present in the human ortholog of the *Drosophila* protein cabut, KLF11. Polyproline-rich regions are *underlined* and denoted in *red*. The A347S variant found in a subset of MODY7 patients is identified by an *asterisk* (*). *B*, cabut and KLF11 were compared utilizing 15-amino acid (*a.a.*) sliding windows across the N terminus of each protein prior to the conserved zinc finger domain and scoring for overall hydrophobicity using the Kyte-Doolittle scale (20); polyproline helix conformations using experimental derived values (21); and overall proline, glycine, and glutamine content (*PGQ* index). The analysis reveals the presence of polyproline-enriched islands amid stretches of strongly hydrophobic residues.

**FIGURE 2.**
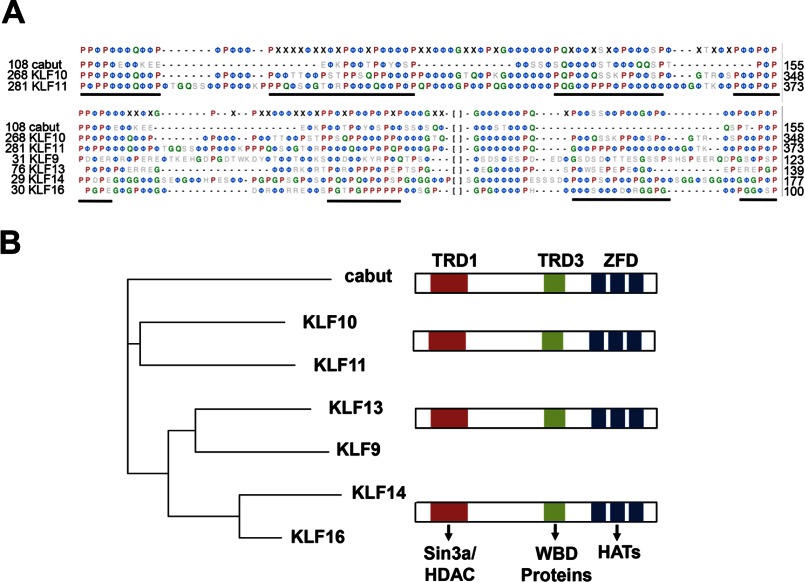
**The KLF11 TRD3 protein domain is conserved in the *cabut* family of metabolic transcription factors.** *A*, a comparison of biochemical-biophysical structure of the proline-rich domain between cabut and KLF10/11, its two closest orthologs, along with their consensus is shown at the *top*. A similar comparison is shown *underneath* that also includes the related basic transcription element-binding protein subfamily of KLF proteins (KLF9, KLF13, KLF14, and KLF16) and the corresponding consensus. Regions with high polyproline content with either high hydrophobic character or high proline, glycine, and glutamine content (PQG index) were deemed putative TRD3 domains. KLF11 contains four distinct PRM domains that are embedded within an environment of hydrophobic amino acids (Φ) and a few flexible residues, such as Gly and Gln (*green* residues). Proline residues are denoted in *red*. Represented regions include amino acids 108–155 for cabut, 268–348 for KLF10, 281–373 for KLF11, 31–123 for KLF9, 76–139 for KLF13, 29–177 for KLF14, and 30–100 for KLF16. *B*, evolutionary analysis reveals high pressure for conservation of this protein domain from *Drosophila* cabut to human with the closest orthologs being that of the transforming growth factor-β-inducible early gene family of KLF proteins (KLF10 and KLF11). Members of the basic transcription element-binding protein family of KLF proteins (KLF9, KLF13, KLF14, and KLF16) are also closely related. *WBD*, WD40 binding domain; *HATs*, histone acetyltransferases; *HDAC*, histone deacetylase; *ZFD*, zinc finger domain.

Previous studies in human populations have demonstrated that defined changes in the sequence of the KLF11 protein (Q62R, T220M, and A347S) associate to early onset type II diabetes mellitus **(**OMIM MODY7) (6). Functional studies demonstrated that these three KLF11 variants have impaired transcriptional activity compared with wild type. Subsequent studies focused on characterizing the mechanism of function for KLF11 Q62R demonstrated that this protein is defective in binding to Sin3a, resulting in lower levels of insulin expression and promoter activity (6). In contrast, here, our structure-oriented bioinformatics analyses demonstrated that the A347S variant falls within the TRD3 of KLF11 and has the potential to disrupt the function of this domain for which cofactors have long remained unknown (Fig. 1*A*). Analysis of the KLF11 TRD3 sequence within the region affected in MODY7 (A347S) as well as the corresponding region of its fly ortholog *cabut* identified several proline-rich motifs (PRMs; Fig. 1*B*). PRMs are present in many transcription factors, chromatin regulators, and epigenetic proteins and facilitate protein/protein interactions (30). Interestingly, we found a high degree of conservation of these PRMs in all KLF proteins, which are orthologs of the fly *cabut* gene, a known metabolic regulator (2). These human proteins form two KLF subfamilies, namely the transforming growth factor-β-inducible early genes (KLF10 and KLF11) and basic transcription element-binding proteins (KLF9, KLF13, KLF14, and KLF16) (1). The PRMs identified in these studies display key characteristics, falling into distinct structural types. Two PRMs mark both the N-terminal (PRM1, PPØPØØØQØØP where Ø represents a hydrophobic residue) and C-terminal (PRM4, GØXXØØPØØPØP) boundaries for the TRD3. Two other PRM domains are located centrally (PRM2, QØØPØPQPØØØGP and PRM3, ØØPPPØPØØØ). All of the PRMs are embedded within a biochemical-biophysical environment entirely provided by hydrophobic amino acids and a few flexible residues such as Gly and Gln. Notably, analyses of extensive available data from NMR, x-ray crystallography, phage display experiments, and liquid phase binding assays demonstrate that PRMs of this type are highly enriched within *bona fide* protein/protein interaction modules (30–32). These repetitive PRMs are shared by several protein/protein interaction modules. Binding of PRMs to their partners is required to form structures that have more stable thermodynamic properties. Therefore, to guide further biochemical studies, we developed a computational model that defines this KLF protein/protein interaction domain (Fig. 2*A*); this model revealed that the TRD3 is located immediately upstream of the KLF DNA binding domain in the region affected by MODY7 A347S. Evolutionary analysis of the TRD3 region further demonstrated the high pressure for preservation of the domain between fly and human across the cabut/KLF family (Fig. 2*B*). Thus, combined, these results support the prediction that the TRD3 domain functions as a proline-rich module that likely mediates protein/protein interactions, a hypothesis that we subsequently tested experimentally.

To define potential binding partners for this protein interaction domain, we utilized both the yeast two-hybrid system and domain-specific protein binding arrays. The bait for yeast two-hybrid studies and the recombinant protein for hybridization to protein arrays were derived from the region of KLF11 encompassing the TRD3, which consists of amino acids 273–351. The advantage of utilizing these two distinct assays was to perform both unbiased (yeast two-hybrid) and candidate-based (protein array) approaches to identify potential TRD3-interacting proteins. For protein array experiments, we used a high TRD3/bound array protein ratio (5:1) so that proteins from the array would capture different amounts of TRD3 according to their binding affinities as detected by different intensities. Arrays also included both positive and negative controls. Thus, the identities in the autoradiographs gave an indication of relative binding strengths. These experiments showed that the KLF11 TRD3 binds to several WD40-containing proteins, including Gβ_1_, Gβ_2_, WD40 repeat domain 6, and echinoderm microtubule-associated protein-like 2 in addition to SH3- (eight positives from a total of 38), WWI- (10 positives from a total of 34), and WWII-containing proteins (four positives from a total of 33) (Table 1). When analyzed, the potential TRD3 interactome defined by both methods reveals a network of proteins that, similar to KLF11, participate in the regulation of transcription. Furthermore, some of these factors, such as ITCH, have previously identified roles in the functional regulation of cabut proteins (KLF10) (33), thus providing internal validation for our experiments. Interestingly, although they exhibit less conservation in overall domain structure, TRD3-like domains are present in a large number of KLF proteins outside of the cabut subfamily (supplemental Table 1), and some have been shown to interact with proline-rich binding proteins, such as Nedd4-like protein WWP1 for KLF2 (34) and Yes-associated protein and WWP1 for KLF5 (35, 36). Thus, from these experiments, we conclude that cabut members as well as other KLF proteins appear to have undergone evolutionary pressure to conserve proline-rich domains that harbor the potential to regulate their function. Combined, these structural relationships led us to perform subsequent studies aimed at clarifying the biochemical properties and functional impact of the TRD3 interactions in the transcriptional regulation of metabolic gene networks, focusing on isolating those that are disrupted by the MODY7 A347S variant.

**TABLE 1:**
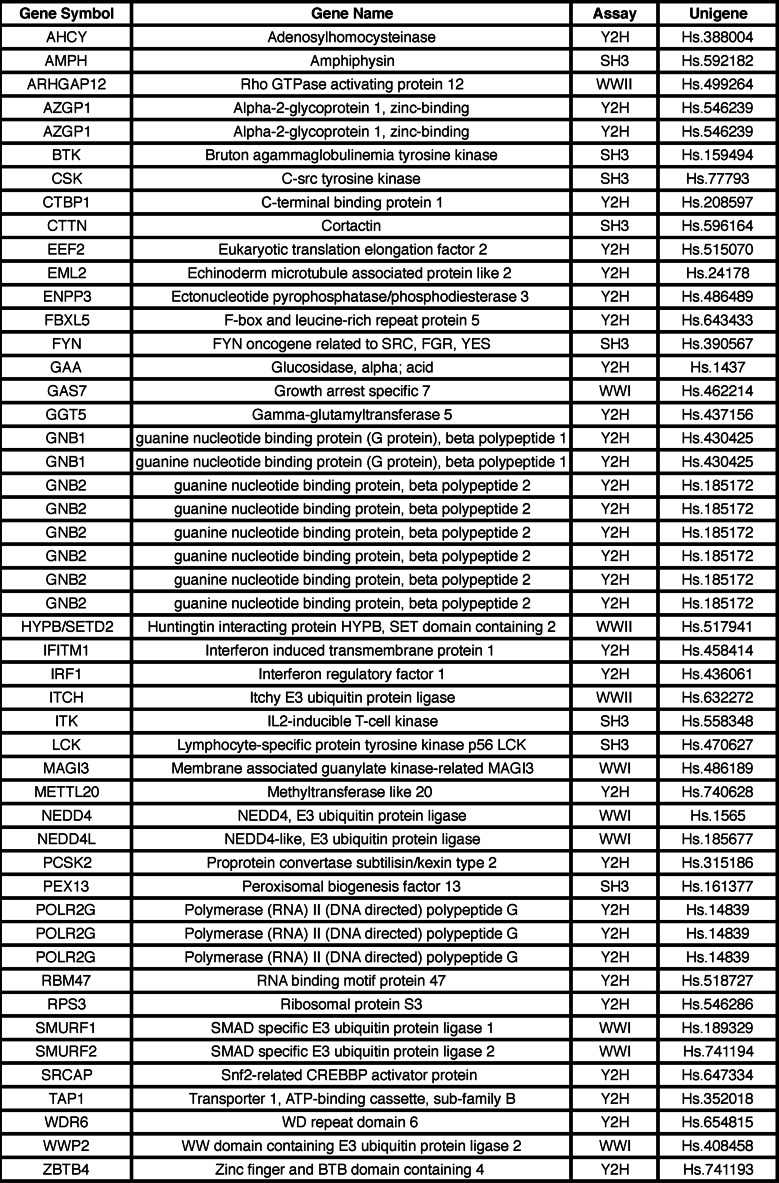
**List of KLF11 TRD3-interacting proteins identified by yeast two-hybrid and protein array experiments** Y2H, yeast two-hybrid; SH3, SH3 protein array; WWI, WWI protein array; WWII, WWII protein array.

##### Membrane-to-nucleus Signaling Pathways Regulate the KLF TRD3 Binding Domain

**FIGURE 3.**
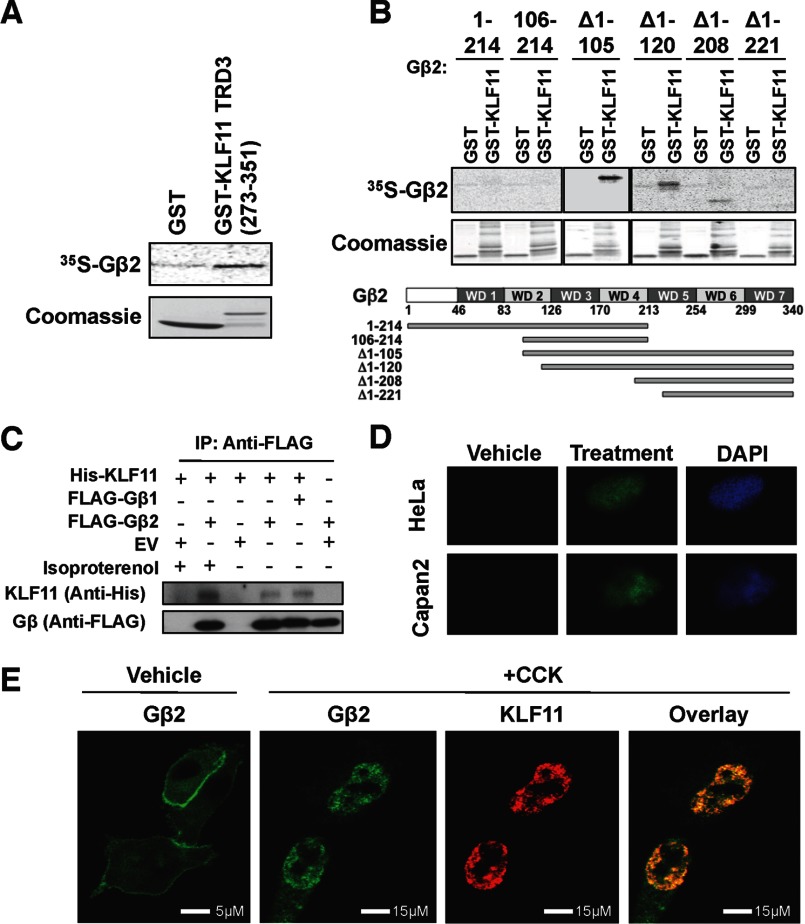
**Membrane-to-nuclear coupling of the KLF11 TRD3 with the Gβ subunit of the heterotrimeric G protein.** *A*, Gβ_2_ binds the KLF11 TRD3 *in vitro*. GST-KLF11 TRD3 (amino acids 273–351) and GST alone were utilized for GST pulldown assays with ^35^S-labeled *in vitro* translated Gβ_2_. Phosphorimaging results of [^35^S]Gβ_2_ binding (*upper*) and Coomassie staining of GST and GST-KLF11 TRD3 proteins as a loading control (*lower*) are shown. GST alone was included as a negative control. *B*, KLF11 binding requires Gβ_2_ WD40 repeats 5–7 *in vitro*. GST-KLF11 full length and GST alone were utilized for GST pulldown assays with various ^35^S-labeled *in vitro* translated Gβ_2_ deletions. The *upper panel* shows phosphorimaging results of [^35^S]Gβ_2_ binding (*upper*) and Coomassie staining of GST and GST-KLF11 proteins as a loading control (*lower*). The *lower panel* depicts a schematic representation of Gβ_2_ with the location of its WD40 repeats as well as the various deletions used for mapping the Gβ_2_/KLF11 interaction. These experiments indicate that Gβ_2_ needs intact WD40 repeats 5–7 for binding to KLF11. *C*, Gβ binds to KLF11 in cells, and this is enhanced by activation of GPCR signaling. HeLa cells were co-transfected with epitope-tagged Gβ and KLF11. Both, FLAG-tagged Gβ_1_ and Gβ_2_ immunoprecipitate (*IP*) His-tagged KLF11. Empty vector (*EV*) was utilized as a negative control in combination with either FLAG-Gβ_2_ or His-KLF11 separately. Experiments were repeated in the presence of isoproterenol, which resulted in a more robust interaction. *D*, BiFC of KLF11 and Gβ_2_ demonstrates nuclear interaction. Cotransfection of N-terminal EYFP protein (EYFP(1)) fused to KLF11 with the C-terminal EYFP (EYFP(2)) fused to Gβ_2_ in both HeLa and Capan2 cells was performed, and subsequently, cells were stimulated with isoproterenol or secretin, respectively. Although control treatment (vehicle) does not show a positive interaction, activation of GPCRs in both cell types (treatment) demonstrates interaction in the nucleus through fluorescence reconstitution and nuclear DAPI co-stain. *E*, GPCR stimulation induces the translocation of Gβ_2_ from the cell membrane to the nucleus where it co-localizes with KLF11. CHO-CCKAR cells were transiently transfected with epitope-tagged Gβ_2_ and KLF11. Without stimulation of CCKAR (*Vehicle*), Gβ_2_ localizes to the membrane and cytoplasm. Upon CCK treatment (+*CCK*), Gβ_2_ (*green*) translocates to the nucleus where it localizes with KLF11 (*red*). *Overlay* shows extensive co-localization (*yellow*) of these two proteins, confirming their close proximity.

For subsequent functional studies, we chose to study Gβ_2_ as a model for a TRD3-interacting protein because this interaction was identified as our most represented candidate via several clones from the yeast two-hybrid system. This is functionally relevant because in this system protein/protein interaction occurs in the eukaryotic nucleus (yeast), the compartment in which KLF11 performs its function. Additionally, the interactions of a few KLF proteins with SH3 and WW domains have been characterized previously (33–37), but those with WD40-containing proteins, such as Gβ (38), remain unknown, increasing the potential novelty of our findings. Furthermore, Gβ is known to regulate cell signaling cascades that are critical for maintaining metabolic homeostasis, including those in diabetes (39–41). In fact, genetic variants of these proteins have also been associated with the development of this disease (39, 42). Previous reports have also shown that the Gβ subunits translocate to the nucleus and interact with other transcription factors, including Fos and the glucocorticoid receptor (GR) (43, 44). Therefore, to confirm the interaction between KLF11 and Gβ_2_ detected by our yeast two-hybrid experiments, we initially performed *in vitro* binding assays using ^35^S-labeled Gβ_2_ and a GST fusion protein of the KLF11 TRD3 (amino acids 273–351). We demonstrated that Gβ_2_ indeed binds the KLF11 TRD3 *in vitro* (Fig. 3*A*). Furthermore, based upon the sequences recovered from the positive yeast two-hybrid clones, we utilized deletions of Gβ_2_ to determine the region of this protein required for interaction with KLF11. These investigations revealed that binding with KLF11 is maintained by Gβ_2_ deletions containing a minimum of amino acids 209–340 as observed by positive GST-KLF11/[^35^S]Gβ_2_ interaction for Gβ_2_Δ1–105, Δ1–120, and Δ1–208 (Fig. 3*B*). However, interaction was not detected with the N-terminal regions encompassing Gβ_2_(1–214), Gβ_2_(106–214), and Gβ_2_Δ1–221 (Fig. 3*B*), which presumably disrupt the protein in WD40 repeat 5. Therefore, Gβ_2_ with intact WD40 repeats 5–7 is necessary for interaction with KLF11.

We next investigated this interaction in cultured cells and whether the KLF11-Gβ_2_ can transduce signals from the membrane to the nucleus. For this purpose, HeLa cells under basal conditions were co-transfected with His-tagged full-length KLF11 and either empty FLAG control vector or FLAG-tagged Gβ_2_. Upon immunoprecipitation of FLAG-tagged Gβ_2_, KLF11 was detected (Fig. 3*C*). This result was further confirmed by immunoprecipitation of FLAG-tagged Gβ_1_ as this isoform of Gβ was also recovered by our yeast two-hybrid assay, which corroborated binding to KLF11 (Fig. 3*C*). To examine the influence of G protein-coupled receptor (GPCR) activation at the plasma membrane by a physiological stimulus, we first utilized isoproterenol, an agonist of β-adrenergic GPCR signaling, which regulates many functions in HeLa cells (45). Interestingly, a more robust immunoprecipitation of KLF11 was observed with Gβ_2_ upon isoproterenol treatment (Fig. 3*C*). To visualize this interaction in living cells, we used BiFC by fusing the EYFP N terminus (EYFP(1)) to KLF11 and the EYFP C-terminal portion (EYFP(2)) to Gβ_2_. These constructs were co-transfected in both HeLa and Capan2 cells and subsequently stimulated with isoproterenol or secretin (46), respectively, for 30 min prior to fixation. Secretin is an agonist for the GPCR family generally with high levels in Capan2 cells (46). Interaction was detected in both cell types upon their respective physiological stimulus as evidenced by yellow fluorescence reconstitution. Fluorescence resulting from the KLF11/Gβ_2_ interaction was localized as expected in the nucleus as determined by nuclear DAPI co-stain (Fig. 3*D*). Co-expression of a negative control leucine zipper protein with either KLF11 or Gβ_2_ fused to their respective EYFP halves did not reconstitute fluorescence, confirming that the fluorescence obtained is specific and devoid of background (data not shown). Representative panels of untreated HeLa or untreated Capan2 cells are also shown (Fig. 3*D*). To complement these studies, we also utilized CHO cells stably transfected with the GPCR CCK receptor A (CHO-CCKAR) (18) and transiently transfected with epitope-tagged Gβ_2_ and KLF11. Similar to the β-adrenergic and secretin receptors, the CCKAR is a well known regulator of metabolic functions (47). In agreement with previous reports, we found that Gβ_2_ localizes to the membrane and cytoplasm in unstimulated cells (Fig. 3*E*). However, upon CCK treatment of the CHO-CCKAR cells, Gβ_2_ translocates to the nucleus where it localizes with KLF11, suggesting that under these conditions they function in close proximity (Fig. 3*E*). Therefore, several physiological stimuli of distinct GPCRs (isoproterenol, secretin, and CCK), which trigger dissociation of β-γ heterodimers from the trimeric G protein α_s_-β-γ complex at the plasma membrane to allow downstream signaling, resulted in interaction between Gβ and KLF11 in the nucleus of three different cell types. Combined, these results not only confirm our yeast two-hybrid experiments but more importantly indicate that activation of GPCRs induces the translocation of the Gβ_2_ subunit, which by forming a complex with KLF11 likely regulates responses (*e.g.* gene expression).

##### Metabolic Gene Networks Requiring Coupling of KLF11 to Cofactors via the TRD3 Are Deregulated by the A347S Variant

**FIGURE 4.**
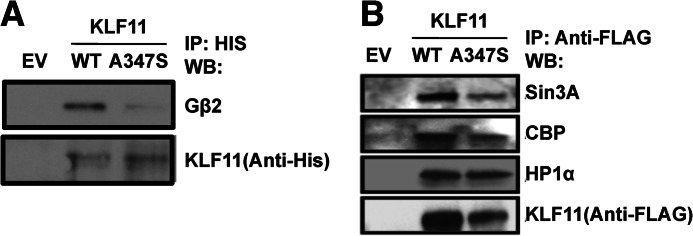
**The KLF11 A347S MODY7 variant disrupts its ability to bind the Gβ subunit.** *A*, the KLF11/Gβ interaction in cells is altered by the A347S variant. Immunoprecipitation (*IP*) of His-tagged KLF11 WT demonstrates binding with endogenous Gβ_2_ in Panc1 cells. However, immunoprecipitation of His-tagged KLF11 A347S shows that this variant disrupts its interaction with Gβ_2_. Empty vector (*EV*) was used as a negative control. *B*, KLF11 A347S does not impair binding to other KLF11 cofactors. Although KLF11 A347S does not interact with Gβ_2_, immunoprecipitation of the His-tagged variant does not affect interaction with other known KLF11 cofactors, namely Sin3a, HP1α, and CBP/p300. *WB*, Western blot.

**FIGURE 5.**
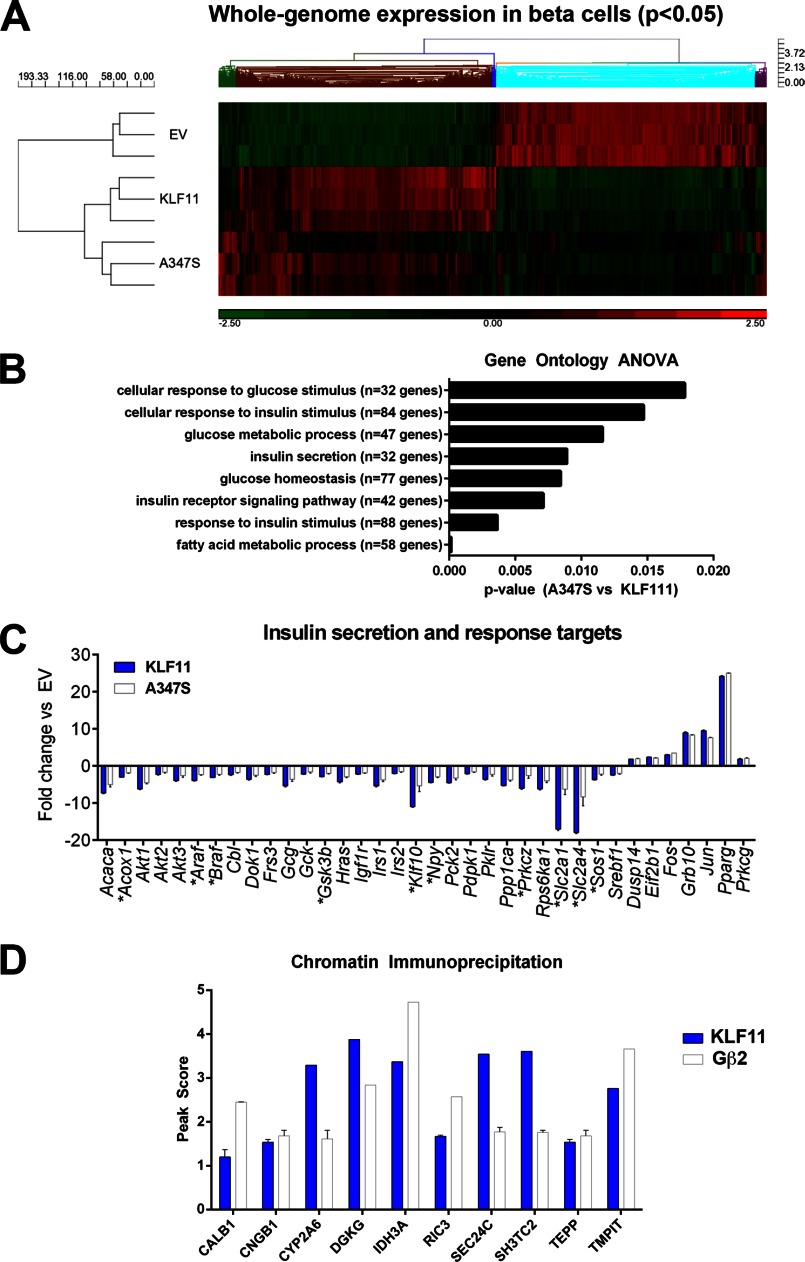
**The KLF11 A347S MODY7 variant deregulates metabolic gene networks that require coupling of KLF11 to cofactors via the TRD3.** *A*, genome-wide query using a whole genome Affymetrix expression profile (Rat Gene 2.0) in pancreatic β cells transduced with empty vector (*EV*), KLF11, or the A347S variant. Significant (*p* < 0.05) genes were clustered to compare the effects of the A347S variant *versus* wild type. A detailed statistical and bioinformatics analysis identified unique genes that significantly associate with KLF11 expression (*p* < 0.05, log_2_ -fold change ±2). This analysis reveals that the targets significantly repressed or activated with wild type KLF11 are altered in the presence of the A347S variant. *B*, gene ontology analysis of variance (*ANOVA*) analysis of KLF11 and A347S targets reveals a distinct enrichment of insulin secretion and response pathway ontological clusters that are significantly disrupted in the presence of the A347S variant. *C*, quantitative PCR validation of a panel of insulin secretion and response genes again reveals the disruptive effect of the A347S variant identified by global expression profiling in *A. Asterisks* represent gene targets where the A347S variant deregulation is significant compared with wild type (*p* < 0.05). *Error bars* represent S.D. *D*, co-occupation of KLF11 and Gβ at human gene promoters. ChIP was utilized to examine co-occupation of KLF11 and Gβ on regions of human gene promoters up to 5 kb upstream of the transcriptional start site. *Error bars* represent false discovery rate on reported peak scores.

Because the region of interaction between KLF11 and Gβ_2_ encompasses the A347S KLF11 variant previously identified in a family of individuals affected by MODY diabetes (6), we examined whether this naturally occurring variant interferes with binding between these two proteins. Immunoprecipitation of KLF11 A347S demonstrated that this variant disrupts its interaction with Gβ_2_ (Fig. 4*A*). Notably, this effect is isolated to binding between KLF11 and Gβ_2_ as the variant did not affect the interaction of KLF11 with its other known cofactors, Sin3a, HP1α, and CBP/p300 (Fig. 4*B*). These results reveal that the KLF11 A347S variant is useful to define whether protein/protein interactions influence the function that KLF11 has in the regulation of metabolic gene expression pathways. Thus, we performed a genome-wide query using a whole genome Affymetrix expression profile (Rat Gene 2.0 ST) in INS-1 rat pancreatic β cells transduced with empty vector control, wild type KLF11, or the KLF11 A347S variant to define genes and in particular metabolic gene networks that are regulated by KLF11 but altered via the A347S variant that interrupts the function of the KLF11 TRD3 (Fig. 5*A*). Clustering of all significantly altered probes (*p* < 0.05) revealed distinct clusters of genes regulated by KLF11. Detailed statistical and bioinformatics analysis identified 2275 unique genes that significantly (*p* < 0.05, -fold change ±2) associate with KLF11 expression, whereas 1205 genes significantly associate with KLF11 A347S expression. Therefore, congruent with its known biological function, these data demonstrate that KLF11 is a regulator of metabolic gene networks and that the A347S variant alters this profile.

Clustering analysis revealed that in β cells expression of the KLF11 A347S variant has an impaired transcriptional regulatory function. To statistically confirm the disruptive effect of the A347S variant on β cell gene expression, gene ontology analysis of variance analysis of the data set was performed. This statistical method demonstrates that KLF11 regulates clusters of genes known to modulate the insulin response pathway, including glucose import, glycolysis, fatty acid metabolism, insulin secretion, calcium and potassium channels, MAPK activity, and PI3K activity among others (supplemental Table 2). Fig. 5*B* displays the overall expression pattern of 32 KLF11-regulated genes with function related to insulin secretion. KLF11 significantly (*p* < 0.05) regulates this insulin-associated gene network, whereas their expression is altered by the KLF11 A347S variant. In addition to this gene network, a subset of other glucose- and insulin-related biological processes significantly deregulated in the presence of the A347S mutant are also shown (Fig. 5*B*). To validate the regulatory effects of KLF11 and the KLF11 A347S variant observed in the genome-wide array, we performed quantitative PCR on INS-1 β cells for which results are shown in Fig. 5*C*. This validation indicates the overall reliability of the array in identifying patterns of gene regulation by KLF11 or its variant. The effects of KLF11 and its A347S variant were also examined in another pancreatic epithelial cell line, Panc1 (supplemental Fig. 1), which also revealed disruption of metabolic pathways associated to the insulin response. Moreover, we also validated the regulatory effect of Gβ_2_ on a subset of identified KLF11-regulated targets in these cells. For this purpose, we performed expression analysis using a construct that targets Gβ_2_ protein to the cell nucleus (Gβ_2_ fused to three copies of the SV40 nuclear localization signal). These experiments demonstrated that transcripts that are significantly regulated by KLF11 are congruently activated or repressed by Gβ_2_ (Table 2), supporting their role in cooperative regulation of these targets. Lastly, using a ChIP assay, we confirmed that human gene promoters can be co-occupied by both KLF11 and Gβ within the first 5 kb of their promoters (Fig. 5*D*). Combined, these data support co-occupancy and regulation of gene targets by this newly identified KLF11-Gβ_2_ transcriptional complex.

**TABLE 2:**
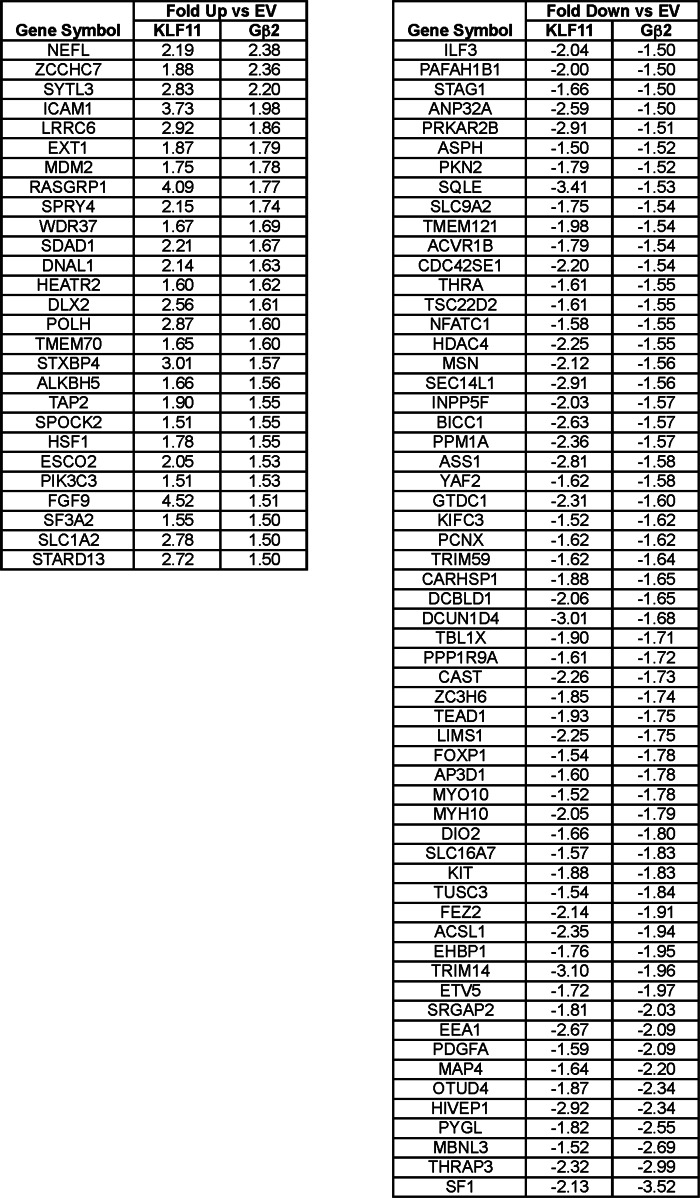
**Gene targets co-regulated by KLF11 and Gβ_2_** Cooperative regulation of targets by KLF11 and Gβ_2_ and the regulatory effect of Gβ_2_ overexpression on a subset of identified KLF11 target genes are shown. Targets significantly regulated by KLF11 demonstrate similar activation or repression in the presence of overexpression of Gβ_2_ with three copies of the SV40 nuclear localization signal. Significance was assumed at *p* < 0.05 and log_2_-fold change ±1.5 for either KLF11 *versus* empty vector (EV) or Gβ_2_ *versus* empty vector.

##### The A347S MODY7 Variant, Which Disrupts Binding to Gβ, Blunts KLF11-mediated Insulin Promoter Activity as Well as Glucose-stimulated Insulin Secretion

**FIGURE 6.**
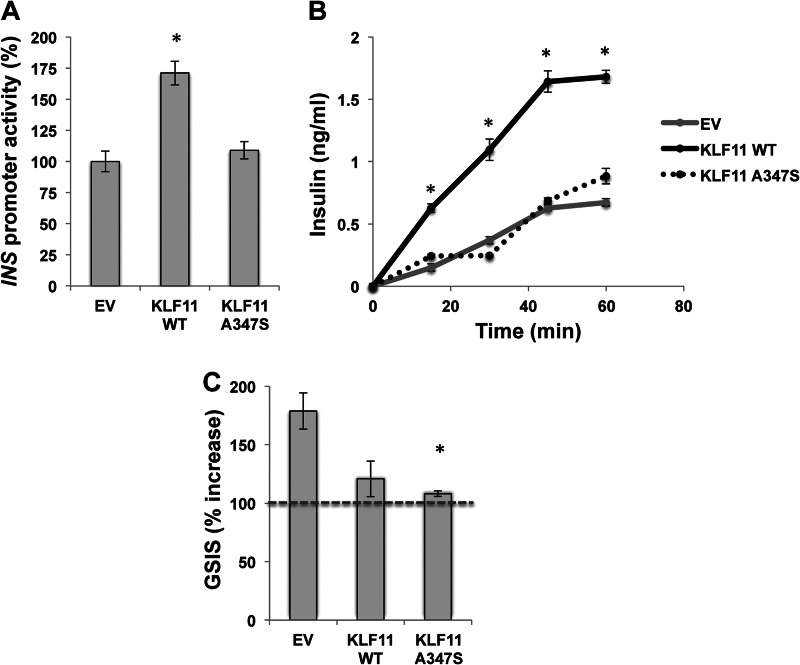
**KLF11-mediated insulin promoter activity and basal insulin levels are altered by A347S combined with blunted glucose-stimulated insulin secretion.** *A*, A347S disrupts KLF11-mediated insulin promoter activity. In INS-1 cells, KLF11 WT increases basal promoter activity (171 ± 9.5%); however, the A347S variant abrogates this effect (109 ± 7.0%). * denotes *p* < 0.005. *B*, levels of insulin secreted from INS-1 cells after infection with adenovirus carrying empty vector (*EV*), KLF11 WT, or KLF11 A347S as measured by ELISA. Forty-eight hours postinfection, glucose was withdrawn by replacing medium with Krebs buffer, and insulin was measured at various time points without glucose. KLF11 WT had significantly higher insulin levels at all time points after glucose withdrawal. * denotes *p* < 0.005. Notably, the A347S MODY7 variant had insulin levels in the same range as the empty vector control, disrupting the KLF11-mediated effect. *C*, glucose-stimulated insulin secretion experiments demonstrate that KLF11 WT results in diminished insulin secretion in response to glucose (120.8 ± 15.3%) compared with empty vector control (178.8 ± 15.5%). The A347S variant significantly reduced the cellular response to glucose (108.1 ± 2.5%). All values were normalized to measurements with no glucose taken just prior to the addition of glucose, depicted by the *broken line* (100%). * denotes *p* < 0.05. *Error bars* in all panels represent S.E.

Previous functional studies have indicated that the three KLF11 MODY7 variants have impaired transcriptional activity compared with wild type (6). The quantitative PCR-validated genome-wide expression analyses reported here on the A347S variant similarly demonstrated impairment of the effect observed for KLF11 wild type (WT). Consequently, we sought to gain insight into the role of this MODY7 variant in the context of β cell function. Therefore, we first tested the effect of the A347S variant on basal insulin promoter activity. Note that increased levels of KLF11 should mimic in part the up-regulation of this gene observed upon glucose stimulation (6). Notably, we observed that although as reported previously (5, 6) KLF11 WT increases basal promoter activity in INS-1 cells (171 ± 9.5%; *p* < 0.005) the A347S variant abrogates this effect (109 ± 7.0%; Fig. 6*A*). Subsequently, we measured levels of insulin secreted from INS-1 cells after infection with adenovirus carrying empty vector, KLF11 WT, or KLF11 A347S by ELISA. Cells were cultured for 48 h postinfection and depleted of glucose by replacing medium with Krebs buffer. Next, we measured insulin secretion after 0, 15, 30, 45, and 60 min of glucose withdrawal. Congruent with the results on *INS* promoter activity shown in Fig. 6*A*, KLF11 WT produced higher insulin levels at all time points upon withdrawal of glucose (*p* < 0.005; Fig. 6*B*). However, when compared with the effect of KLF11 WT, the A347S MODY7 variant showed an impaired response with insulin levels in the same range as the empty vector control (Fig. 6*B*). As expected from a glucose-inducible activator of the insulin gene, KLF11 indeed increased the total levels of this hormone to a degree that could not be further stimulated by glucose (120.8 ± 15.3% *versus* no glucose) when compared with empty vector control (178.8 ± 15.5% *versus* no glucose; Fig. 6*C*). In contrast, cells transduced with the A347S variant showed low levels of insulin secretion even in the presence of glucose (108.1 ± 2.5% *versus* no glucose; *p* < 0.05). Thus, combined, these data demonstrate that although enhanced levels of KLF11 WT appear to recapitulate the glucose response by increasing basal insulin levels and promoter activity the MODY7 A347S variant is impaired in these functions.

## DISCUSSION

Although the strength of modern genetics is to discover the association of genes and their variants to disease with a high level of resolution, significantly less is known regarding how the protein products work to give rise to diseases. This has been the case for those genes within the KLF family of transcriptional regulators, such as KLF11 and KLF14, which have been strongly associated to diabetes, obesity, and insulin resistance/metabolic syndrome (5, 6, 48–50). For instance, very little is known regarding how these genes influence metabolic function. The current study provides mechanistic insights into how human diabetes-associated genetic mutations and variants that occur in these metabolic transcription factors, such as the KLF11 A347 variant, impair the regulation of metabolic gene networks. In addition, we provide biochemical and genetic evidence for the existence of a novel transcriptional regulatory pathway that appears to translate membrane-to-nuclear signals, such as those imparted by signaling proteins, in particular the Gβ subunit, to the KLF11-mediated regulation of gene expression patterns relevant to metabolism and diabetes.

The functional importance of the region corresponding to the KLF11 TRD3 was suggested in 1999 through a careful mapping of the N-terminal domain of this protein (15), although the identity of a potential cofactor for this domain has remained elusive. Thus, the identification of the TRD3 as a protein interaction domain has increased our mechanistic understanding of how KLF11 regulates its functions and how the MODY7 A347S variant impairs it. The TRD3 functions as a protein interaction module as demonstrated by yeast two-hybrid, protein array, immunoprecipitation, and BiFC experiments. In addition, we confirmed that the Gβ subunit of the heterotrimeric G protein, the most represented candidate from our yeast two-hybrid screening, interacts with KLF11 via its TRD3 domain. Furthermore, we demonstrated that the MODY7 A347S variant disrupts binding to this protein. In addition, using a solid phase *in vitro* binding assay (protein array), we showed that the TRD3 domain can bind to several types of Pro-rich binding modules, such as WWI, WWII, and SH3 domain-containing proteins. Interestingly, some of these proteins, such as ITCH and Nedd4-like proteins, have been shown previously to bind to KLF transcription factors (33–35) at their C-terminal domains, providing a cross-validation of our experiments. Although these regions show less homology to the corresponding cabut-like TRD3 domain, nevertheless they display the structural and functional characteristics that suggest that binding to members of these families of proteins immediately upstream of the KLF DNA binding domain is a wider mechanism of regulation for KLF transcription factors.

The TRD3-binding proteins identified here are a wide variety of eukaryotic proteins that have a range of functions, including as adaptor/regulatory modules in signal transduction and in pre-mRNA processing, cytoskeleton assembly, gene transcriptional activation, and cell cycle control. Of further interest, Gβ is a WD40 repeat protein, a large protein family for which members are increasingly being recognized as key regulators of chromatin dynamics, transcription, and epigenetics (51, 52). Among the diversity of WD40 domain functions, a common theme has emerged that they collude as repeats to form β-propeller structures that act as a platform for the stable or reversible association of binding partners. For instance, WD40 domains are overrepresented in histone deacetylase and histone methyltransferase complexes with well known examples found in Groucho, Polycomb group protein EED (embryonic ectoderm development), and WDR5 (51, 52). The Gβ subunits, which here have been associated to KLF11, have also been reported to interact with other transcription factors, including Fos and the GR (43, 44). In fact, analyses of the GR (amino acids 263–419) and Fos (amino acids 159–238) regions known to interact with Gβ reveal remarkable similarities to the KLF11 TRD3 in that they contain proline-rich domains embedded within a sequence that is rich in hydrophobic as well as flexible amino acids (Gly and Gln). The WD40 motif of Gβ may be essential for the GR interaction as a Gβ structural analog, RACK1 (receptor for activated protein kinase C 1), also binds GR (53). This sequence feature is another similarity between how Gβ works with this steroid receptor and KLF11. For example, activation of G_i_-coupled somatostatin receptor in rat pituitary GH3 cells induces its translocation to the nucleus where it works as a corepressor through direct binding to the AF-2 domain of GR (43). Similarly, we showed that the activation of the β_2_-adrenergic receptor by isoproterenol or the secretin receptor by secretin induces the formation of the KLF11-Gβ_2_ complex in the cell nucleus. Together, these results suggest that members of very distinct families of transcription factors can utilize the Gβ_2_ subunit to mediate their function in gene expression.

It is important to discuss how, when combined, the molecular and cellular data reported in this study contribute to a better understanding of the mechanisms of insulin secretion and its potential alterations in MODY7. The current model for KLF11 function in β cells indicates that glucose enhances the intracellular levels of this transcription factor, which in turns binds to the −331 site within the insulin gene and activates the synthesis of this hormone (5, 6). Thus, although glucose stimulates insulin secretion in cells transduced with empty vector control virus, KLF11 WT increases the total levels of insulin to a degree that cannot be further stimulated by glucose. On the other hand, the KLF11 A347S fails to achieve the same stimulation of insulin secretion whether in the absence or presence of glucose. Therefore, these results indicate that β cells carrying the A347S MODY7 variant are impaired in producing normal levels of insulin even after treatment with physiological stimuli. Furthermore, our data show that insulin is not the only gene regulated by KLF11. Like other diabetes-associated transcription factors, KLF11 regulates gene networks that are involved in metabolism that are also affected by the A347S variant. We believe that these alterations can also compromise the function of β cells as well as those of target tissues in which KLF11 is highly expressed. Thus, disruption of the newly characterized KLF11 regulatory domain (TRD3) as in MODY7 has wider effects on gene expression than anticipated, helping to clarify disease mechanisms.

The observations described here are also of significant biological importance because the role of Gβ_2_ in the nucleus has remained elusive for many decades. Fortunately, the working model that emerges by the congruent association of all data described in this study as well as those available for the GR is that extracellular signals induce the binding of WD40 proteins to these transcription factors to regulate metabolic gene networks. Consequently, disruption of this mechanism should lead to alteration in the regulation of these networks, an effect that we observed experimentally and that is congruent with a role of MODY7 A347S in human diabetes.

In summary, the current study extends the available knowledge on KLF proteins and identifies a novel evolutionarily conserved protein/protein interaction domain that is involved in the transcriptional regulation of metabolic gene networks. The biochemical contribution of this work lies in the extensive characterization of this domain, identification of Gβ as a binding partner, and the role of this domain in the regulation of metabolic gene networks. Lastly, the fact that mutations in this pathway that are known to cause diabetes impact the regulation of metabolic gene networks should be taken into consideration for understanding potential molecular mechanisms that contribute to disease phenotypes.

## Footnotes

3 The abbreviations used are:

KLF: Krüppel-like factor
MODY: maturity onset diabetes of the young
TRD3: transcriptional regulatory domain 3
GPCR: G protein-coupled receptor
CCK: cholecystokinin
CCKAR: CCK receptor A
EYFP: enhanced YFP
CBP: cAMP-response element-binding protein (CREB)-binding protein
SH3: Src homology 3
BiFC: bimolecular fluorescence complementation
PRM: proline-rich motif
GR: glucocorticoid receptor.
